# 

**Published:** 1897-07

**Authors:** 


### MISSISSIPPI VALLEY MEDICAL ASSOCIATION.

The next meeting of the Mississippi Valley Medical Association will be held in Louisville, on Oct., 5, 6, 7 and 8, 1897. All railroads will offer reduced rates.

The President, Dr. Thos. Hunt Stucky, and the Chairman of the Committee of Arrangements, Dr. H. Horace Grant, promise that the meeting will be the most successful in the history of the Association, and this promise is warranted by the well-known hospitality of Louisville and Kentucky doctors.

Titles of papers should be sent to the Secretary, Dr. H. W. Loeb, 3559 Olive Street, St. Louis.

